# Functional non-parametric mixed effects models for cytotoxicity assessment and clustering

**DOI:** 10.1038/s41598-023-31011-1

**Published:** 2023-03-11

**Authors:** Tiantian Ma, Dan Richard, Yongqing Betty Yang, Adam B Kashlak, Cristina Anton

**Affiliations:** 1 Mathematical and Statistical Sciences, University of Alberta, Edmonton, Canada; 2 Mathematics and Statistics, Grant MacEwan University, Edmonton, Canada

**Keywords:** Cell biology, Chemical biology, Statistics, Data mining, Functional clustering, Machine learning, Statistical methods

## Abstract

A multitude of natural and synthetic chemicals are present in our environment.Through the study of a compound’s cytotoxicity, researchers can carefully set regulations regarding how much of a certain chemical in the ambient environment is tolerable. In the past, research has focused on point measurements such as the LD50. Instead, we consider entire time-dependent cellular response curves through the application of functional mixed effects models. We identify differences in such curves corresponding to the chemical’s mode of action—i.e. how the compound attacks human cells. Through such analysis, we identify curve features to be used for cluster analysis via application of both k-means and self organizing maps. The data is analyzed by making use of functional principal components as a data driven basis and separately by considering B-splines for identifying local-time features. Our analysis can be used to drastically speed up future cytotoxicity research.

## Introduction

Chemical compounds naturally present in the environment or produced by industrial processes can be detrimental to human cells, so it is important to develop efficient methods to study cytotoxicity. Cell based in vitro assays are commonly used for automated toxicity screening of large chemical libraries. Since time has a key role in analyzing a chemical’s effect on cellular response, in this paper we apply tools from functional data analysis^1–3^ to study experimental data obtained from the Alberta Center for Toxicology Cytotoxicity Profiling Project with the dual goals of modeling the growth and death of cells exposed to each chemical and clustering chemicals that attack human cells in a similar fashion.

**Figure 1:**
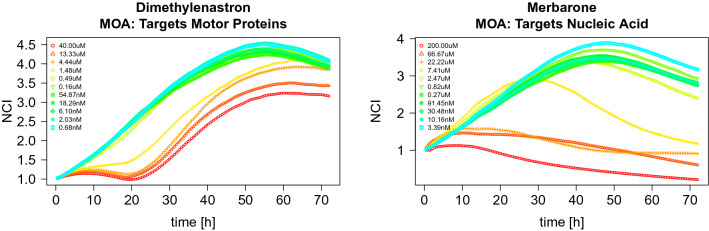
Examples of normalized data TCRCs from two chemicals with different MOAs and 11 concentration levels.

In the initial experiment, a total of 63 chemical compounds were tested at 11 different chemical concentration levels and time-dependent cellular response curves (TCRCs) were collected for each chemical to record the evolution of the Cell Index (CI), which closely reflects not only cell growth and cell death, but also cell morphology. These along with 12 control samples with no chemicals applied give a total of $11\times 63+12=705$ curves in the data set. Each chemical can be categorized by its mode of action (MOA), which is the characteristic set of physiological and behavioural responses resulting from chemical-target action that describes an adverse biological response such as changing cell population, cell morphology, and cellular functions. Two examples of sets of 11 TCRCs from different MOA categories are displayed in Fig. 1. A primary goal of cytotoxicity research is to identify differences between the MOA categories. We approach this goal of identification by analyzing the patterns of TCRCs. Based on known biological and chemical characteristics, the 63 chemicals used in this experiment are divided in 10 MOA categories^4^ (see Table A.1 in the appendix for more details).

This specific set of experimental data has been considered in multiple prior articles^4–9^ and is available from the authors on request. In^5,6^ mathematical models based on differential equations capable of reproducing the TCRCs are constructed and the effects of chemical compounds on the survival/death of the cells’ population are investigated. In^7,8^, the TCRCs are used to extract features about the toxicity potency of the chemicals, such as the LD50—i.e. the chemical concentration leading to the death of 50% of tested cells—or the area under the normalized TCRCs. After transforming the TCRCs data using principal component analysis, Xi et al.^9^ uses a model-based hierarchical clustering algorithm. However, the identified clusters are not identical to the MOA categorization. Classification based on neural networks and support vector machines are successfully applied in^4^ with training sets given by the data in the 10 groups based on MOA. However, their chosen tools lack model interpretability and takes a supervised as opposed to unsupervised learning approach. Further analysis and statistical testing is needed to explain how the different MOA categories translate into different characteristics of the TCRCs.

**Figure 2:**
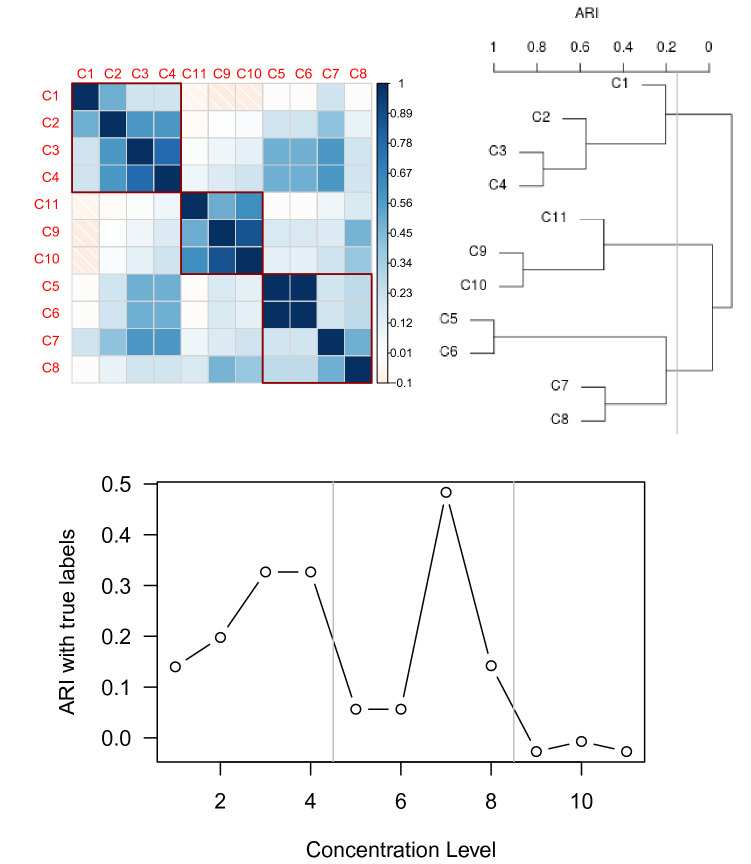
On the top left is a plot of ARI values comparing pairwise k-means clustering similarity between the 11 concentration levels. The red boxes indicate hierarchical clustering of the concentration levels based on ARI using complete linkage. On the top right, is the cluster dendrogram corresponding to the ARI value. The bottom plot charts the ARI values for similarity between k-means cluster assignment and the true labels.

It is not obvious by visual inspection how to uncover from the TCRCs the 10 groups based on MOA because compounds with different MOA may have similar profiles (see Fig. 2 in^4^ showing resembling TCRCs from four different MOA groups). In this paper, we approach the TCRC dataset via functional data analysis. We fit two functional linear mixed effects models (FLMMs), constructed using functional principal components and cubic B-splines, and we use these models to analyze the differences between the characteristics of the TCRCs corresponding to different MOA groups. Moreover, based on the coefficients of the FLMMs we apply several approaches to cluster this experimental data with respect to cytotoxicity. Since the number of chemical substances used in today’s society is continuously increasing, it is very important to develop statistical methods based on multi-concentration TCRCs to screen chemical compounds with unknown MOA and classify/cluster them.

The main difficulty to clustering functional data is that the data belong to an infinite dimensional space. Thus, the idea for many methods for clustering functional data is to approximate the data into a finite basis of functions or using some dimension reduction techniques such as functional principal component analysis^3^, and then to use a clustering method for finite dimensional data (see^10^ for a survey about functional data clustering). Here we consider wavelets, functional principal component analysis (fPCA), and cubic B-splines bases and we apply the k-means and self organizing maps (SOMs)^11^ clustering algorithms.

More recent research on clustering functional data includes^12,13^ among others. The latter of which considers similar mixed models but for non-Gaussian data. It estimates a transformation function to “normalize” and fits a mixture model via a penalized EM algorithm. In contrast to this and similar mixture model approaches, we take a two-step approach where a functional random effects model is first fit to the data allowing for projection of the data into finite dimensions. Then, post-hoc multivariate clustering is applied to projected curves. This allows us to account for concentration-based fixed and random effects before clustering chemicals by their MOA group. The data considered in^13^ consists of three groups of patients with no, early, and regular Alzheimer’s disease. Our data consists of 10 groups of chemicals with TCRCs for 11 different chemical concentration levels each. Hence, our dataset has the added complication of each chemical having a collection of observed curves associated with it and a varying concentration parameter. See the tables in the supplementary material for more details on the chemicals and concentration levels tested.

Wavelet coefficients were used in^4^ for classification of the same TCRC dataset, and in^14^ a k-centroid clustering algorithm is constructed using dissimilarity measures based on wavelet-coherence tools and is applied to French electricity power demand functional data. We apply k-means and SOMs clustering algorithms to the wavelets coefficients, and we analyze the influence of various parameters on the clustering performance. Projecting onto a B-spline basis or doing fPCA are some of the most popular methods used in the first step for clustering functional data (see the citations in^10^). Here we have a somehow different and more difficult problem because for each chemical we have 11 curves representing the evolution of the number of cells after exposure to the chemical at 11 different concentrations. Our approach is to include the B-spline basis projections and the fPCA in the context of the FLMMs.

After the data is presented in section “Cytotoxicity data: overview”, we include the methodology in section “Methodology”. The application of FLMMs to the cyto-toxicity data is illustrated in section “Application to the cyto-toxicity data”. Two approaches to FLMMs are considered: fPCA and cubic B-splines. In section “Cluster analysis”, clustering results are presented from both the k-means and SOMs algorithms.

## Cytotoxicity data: overview

The data set in this study were generated from the in-vitro assay -xCELLigence Real-Time Cell Analysis system (RTCA)^7^ developed by ACEA Biosciences Inc. (San Diego, USA). This system utilizes 384-well electronic plates (E-Plates 384), on the bottom of which the electric current is impeded by cells attached to electrodes. The impedance data is recorded as direct measurement of cellular status in real time reflecting cell number, cell morphology (attachment and spreading/shrinking), and cell adhesion^15^. The data is converted from impedance to Cell Index (CI) by the following formula^16,17^

$$\begin{aligned} CI = \underset{i=1,\ldots ,I}{{\text {max}}} ({R_{cell}(f_i)}/{R_b(f_i)}-1) \end{aligned}$$

where $R_{cell}(f_i)$ and $R_b(f_i)$ are the frequency dependent electrode impedance with and without cells in the well respectively, and *I* is the number of frequency points $f_i$ at which the impedance was measured.

Human hepato-carcinoma cells line-*HepG2* (ATCC, cat. no. HB-8065) were grown and tested in EMEMbasal media supplemented with 10% fetal bovine serum. All growth and assay were conducted in $37\,^\circ$C tissue culture hood with 95% humidity and 5% CO$_2$.

Without exposure to the test substances, a typical cell growth curve in the negative control of cell culture system exhibits four phases: the lag, the log, the plateau, and the decline phases. The lag phase is the phase when the cells do not divide and adapt to the culture conditions. In the log phase the cell density increases exponentially, until it reaches a steady-state level in the plateau phase. Finally, in the decline phase, the cell death predominates and the number of viable cells declines due to the natural path of the cell cycle and depletion of the nutrient supplements.

The cells were seeded into the wells of E-Plates and grew for approximate 24 h to attach to the bottom surface of the wells before adding test substances. The test substances were added in the log cell growth phase when the cells are the most viable. The chemicals with 11 concentrations (1:3 serial dilution from the stock solution) were dissolved in the cell culture medium in a non-toxic solvent (DMSO), which was chosen due to it being a well documented substance that does not react with the cell culture medium. Negative controls were presented in each E-Plate 384, as the cells in these wells were treated with only assay buffer for substance dilution, but no testing substances. Four replicates of the negative control and the DMSO were considered, and the median values from the replicated experiments were calculated^8^.

Since the TCRCs do not provide much information of MOA before chemicals are added, they are truncated such that only the data after treatment were kept. Furthermore, different TCRCs can have different CI values at 24 h. We focus on the cellular response to testing chemicals, so CI differences from chemical adding and growth variations were minimized by using the Normalized Cell Index (NCI), which is given in^4^ by

$$\begin{aligned} NCI[k] = {CI[k]}/{CI[0]},\quad k=1,2,\ldots ,K \end{aligned}$$

where k refers to different time points after testing chemical addition, and $k=0$ refers to the time point right before treatment ( i.e 24 h after seeding the cells). Using NCI also ensures that all TCRCs start from the same value of one. We consider only the NCI data after chemical treatment.

The cell index is not measured uniformly in time. As in^4^ and^6^ we use cubic smoothing splines to interpolate the experimental data and place all samples on a common uniform time grid prior to statistical analysis (see pp. 594 in^6^ for an illustration of the approximation accuracy). We can apply the methods based on fPCA and cubic B-splines bases to irregularly spaced data^18^, but we need a uniform time grid to determine the wavelet coefficients and we have decided to use the same data set for all the work presented in this paper. The time interval is 1 h for the interpolated data set, so we have observations corresponding to 72 h^4^.

The 63 chemicals are grouped in 10 distinct MOA groups with a highly imbalanced distribution. MOA group 1 (DNA/RNA-Nucleic Acid Targets) and MOA group 10 (Protein- Motor Targets) contain more than half of the 63 compounds with 20 chemicals in MOA 1 and 13 in MOA 10, whereas groups 5, 7 and 9 contain only 3, 2 and 1 compounds, respectively. To make the statistical learning task tractable, we focus our analysis on the two largest MOA groups, 1 and 10. For the sake of cluster analysis, we also combine the remaining 30 chemicals into a third remainder group. The entire list of 63 chemicals partitioned by MOA groups can be found in Table A.1 in the appendix.

### Binning concentration levels

The eleven concentration levels associated with the TCRCs were chosen with some trial and error on the part of the experimenters as it was not clear a priori what amount of each chemical of interest would constitute a small or large dose. The largest dose was chosen to kill off most of the cells and then was diluted to 1/3 the strength 10 successive times to get the 11 concentration levels. However, by merely looking at the resulting data it is evident that some concentration levels produce roughly the same curves with only slight variation. To reduce our concentration factor from eleven levels to a more manageable three, we apply the following data driven binning strategy. Moreover, having binned concentration levels gives us enough replicates to achieve efficient estimates of the concentration-specific functional random intercept covariance structure for the FLMMs presented in section “Application to the cyto-toxicity data”.

Considering only the two largest MOA groups totalling a sample of size 33 as noted above, we apply the discrete wavelet transformation (DWT). Clustering based on the DWT is motivated by the prior work of^4^. By recursively applying wavelet transforms, it leads to multi-level wavelet decomposition, and as in^4^ we extract the wavelet coefficients at the 4th level decomposition.

Next we apply functional k-means clustering to the scaling coefficients via the kmeans.fd() function in the fda.usc R package individually for each of the eleven concentration levels. The result is 11 sets of binary labels. Following that, each of the $11\atopwithdelims ()2$ label pairings can be compared via the Adjusted Rand Index (ARI), a standard similarity measure on labelings^19^. The result is displayed in Fig. 2. Further application of hierarchical clustering using complete linkage finds three clusters of similar concentration levels being $\{C_1,C_2,C_3,C_4\}$, $\{C_5,C_6,C_7,C_8\}$, and $\{C_9,C_{10},C_{11}\}$. Thus, we proceed with a three-level concentration factor with $\text {high}=\{C_1,C_2,C_3,C_4\}$, $\text {medium}=\{C_5,C_6,C_7,C_8\}$, $\text {low}=\{C_9,C_{10},C_{11}\}$ as $C_1$ denotes the highest chemical concentration and $C_{11}$ the lowest. Hence, in what follows, TCRCs from different concentrations from the same binned level will be considered as replicates.

Figure 2 also displays the ARI value comparing the functional k-means labels to the true MOA labels. We note that concentration levels 1 through 8 considered individually yield mediocre performance whereas levels 9, 10, and 11 have extremely poor performance. A main contribution of this work is finding the right way to combine the information present in each of the concentration levels to improve the clustering accuracy.

Other wavelets coefficients were also used for clustering, but the 5th level decomposition was too far reduced to give sensible results whereas level three gave similar results to level four. Instead of projecting into a wavelets basis, we also considered using functional principal components analysis to project the MOA curves onto the four eigenfunctions with largest eigenvalues. Then, the low dimensional data was clustered via the standard k-means algorithm. The resulting clusters were as above except that $C_9$ was left as a singleton. Hence, it is still reasonable to bin the concentration levels as before choosing to include $C_9$ with $C_{10}$ and $C_{11}$.

## Methodology

In this section we present the models, the estimation, and the clustering methods used in this paper. The functional nonparametric mixed effect model is considered in^20^. In that work, estimation of the model is detailed within the framework of Reproducing Kernel Hilbert Spaces. Posterior means and variances are derived, and an EM algorithm is proposed for clustering heterogeneous functional data under this model.

The functional nonparametric mixed effect model from^20^ takes the form

1 $$\begin{aligned} Y_i(t)= \mu (t,{\textbf {x}}_i)+{\textbf {z}}_i^T{\textbf {U}}(t)+\epsilon _i(t),\qquad i=1,\ldots ,n,\ t\in {\mathcal {T}} \end{aligned}$$

with covariate vector ${\textbf {z}}_i$, population mean $\mu (t,{\textbf {x}}_i)$ assumed to be a smooth mean function dependent on scalar and/or functional covariates ${\textbf {x}}_i$, vector of functional random effects ${\textbf {U}}(t)$ assumed to be a vector-valued zero mean square integrable random process on ${\mathcal {T}}$, and iid Gaussian white noise process $\epsilon _i(t)$ along ${\mathcal {T}}$ with variance $\sigma ^2$. Furthermore, ${\textbf {U}}(t)$ is independent of $\epsilon _i(t')$ for all *i*, *t*, $t'$. The functional random effects (FREs) typically include a smooth error term which is a functional random intercept (FRI) with a special structure that captures deviations from the mean function which are correlated along ${\mathcal {T}}$^21^.

Model 1 is a piecewise model, whose vector of FREs ${\textbf {U}}(t)$ is divided into *G* independent blocks, one for each grouping factor—i.e. ${\textbf {U}}(t)=[{\textbf {U}}_1(t)^T,\ldots ,{\textbf {U}}_G(t)^T]^T$. For each factor, different levels are represented by $L_{U_g}$ independent copies ${\textbf {U}}_{gl}(t),l=1,\ldots ,L_{U_g}$. Each of the independent copies consists of $P_{U_g}$ FREs yielding $g^{th}$ block ${\textbf {U}}_g$. Thus, the total number of FREs in the model is $\sum _{g=1}^GL_{U_g}P_{U_g}$. It is assumed that FREs in different blocks are independent and copies within each block are also independent. Only FREs of the same copy are correlated. Accordingly, the covariance of ${\textbf {U}}(t)$ is a diagonal block matrix that has the following form

$$\begin{aligned} C_U(t,t')=\text {diag} [ \underbrace{C_{U_1}(t,t'),\ldots ,C_{U_1}(t,t')}_{L_{U_1} \text {copies}},\ldots , \underbrace{C_{U_G}(t,t'),\ldots ,C_{U_G}(t,t')}_{L_{U_G} \text {copies}}] \end{aligned}$$

where $C_{U_g}(t,t')=Cov [{\textbf {U}}_{gl}(t),{\textbf {U}}_{gl}(t')]$ is a $P_{U_g}\times P_{U_g}$ covariance matrix for $l=1,\ldots ,L_{U_g}$.

Here we consider two approaches to implement the model (1): functional principal components analysis (fPCA) and B-splines. Functional principal components analysis (fPCA) is a standard tool in the analysis of functional data and it is discussed in many textbooks and articles^1,22,23^. Let $X(t)\in L^2({\mathcal {T}})$ be a square integrable random process. For simplicity, assume *X*(*t*) is centred. i.e. $\mu (t) = E[X(t)] =0$. We define

$$\begin{aligned}&K(s,t)= E[X(s)X(t)] =\sum _{k=1}^\infty \lambda _k \xi _k(s)\xi _k(t)&\text {(auto-covariance function)} \\&C(t) = [Ky](t) = \int _{{\mathcal {T}}} K(s,t) y(s) d s,\quad y\in L^2({\mathcal {T}})&\text {(covariance operator)}, \end{aligned}$$

where $\lambda _1\ge \lambda _2\ge \cdots$ are the eigenvalues and $\xi _1$, $\xi _2$, $\ldots$ are the orthonormal eigenfunctions of the covariance operator. The covariance operator is a linear mapping of *y*(*t*) from $L^2$ to $L^2$ which is positive definite and trace class. It is usually assumed that the auto-covariance function *K* can be well approximated by the first few terms in the eigendecomposition. By the Karhunen–Loeve expasion (KL expansion)^1^ the zero mean stochastic process *X*(*t*) can be expressed in the eigenbasis as:

$$\begin{aligned} X(t)=\sum \limits _{k=1}^{\infty }f_{k}\xi _k(t) \end{aligned}$$

where the principal components $f_{k}=\int _{{\mathcal {T}}}\xi _k(t) X(t)dt$ are uncorrelated zero mean random variables with variance $\lambda _k$.

We use denseFLMM R package to fit the model (1). Estimation and prediction in the package denseFLMM are conducted by expanding FREs in functional principal component bases.

We also consider Model 1 projected onto a B-spline basis. The fixed effects and random effects are represented as the linear combination of spline basis functions, and we estimate the coefficients using the package lme4 in R.

We use the functional mixed effects models from above to project onto a finite basis in order to proceed with multivariate clustering. The two methods we will apply for clustering are the classical k-means algorithm and the more general Self Organizing Maps (SOMs) from^11^.

Self organizing maps generalize the k-means paradigm by allowing for a network of nodes that tend towards cluster centers as the algorithm iterates. We use the implementation in the kohonen R package. This method is quite powerful, but it does require the user to choose various settings before running the SOMs algorithm. These include four choices: a neighbourhood function acting as a kernel to determine proximity with Gaussian or Bubble as selections; a topology for the graphical network being either a rectangular or hexagonal grid; a structure being either planar or toroidal; and a grid size indicating the number of nodes in the network. As discussed in the next sections, the initial choices can strongly affect the performance of the SOMs algorithm.

## Application to the cyto-toxicity data

For each of the 63 chemicals in our dataset, there are 11 recorded TCRCs partitioned as discussed above into three concentration levels denoted 1 = high, 2 = medium, 3 = low. We also have baseline curves with no added chemicals (the negative control) corresponding to a concentration level of *none*. We will analyze this data via functional mixed effects models with the concentration factor being treated as a 4-level fixed effect while the chemical factor is treated as a random effect. Within each chemical-concentration combination, we have 3 or 4 replications.

For the TCRCs data, the FLMM here is a hierarchical model that includes a fixed effect for concentration. The three-level concentration factor and the repeated observations are nested within the chemical factor. The remaining hierarchy levels are accounted for by including a random intercept. The model is in the following form

2 $$\begin{aligned} Y_{\tau co}(t) = \mu _{\tau }(t) + B_{\tau c}(t) + E_{co}(t) + \epsilon _{\tau co}(t) \end{aligned}$$

with concentrations $\tau$, chemicals *c*, and replicate *o*. $Y_{\tau co}(t)$ represents the NCI at time point *t*, concentration $\tau$, replication *o*, chemical *c* and $\mu _{\tau }(t)$ is the fixed effect for concentration $\tau$. $B_{\tau c}(t)$ is a concentration-specific functional random intercept for chemical *c*. $E_{co}(t)$ and $\epsilon _{\tau co}(t)$ are a smooth error term and white noise measurement error, respectively. We assume that $B_{\tau c}(t)$ and $E_{co}(t)$ are mutually uncorrelated random processes with zero mean. Since the point-wise variations of concentrations differ from each other, we allow that the covariances of the chemical effects are different for each concentration, which is termed as a concentration-specific FRI. We assume the smooth error $E_{co}(t)$ does not depend on concentration, thus is not concentration-specific.

### The fPCA model

We expand the FREs in the model (2) in functional principal component bases and we use denseFLMM R package to fit the model. To compare the MOA groups 1 and 10 in the TCRCs data-set we start by applying the model (2) separately to the data in each group. We notice that many of the fPCs at different concentration levels are similar. The first and dominant fPC of each concentration is simple in structure, while the second and third fPCs have higher order. More difference occurs in third fPC, especially for low concentration, where the fPC in MOA group 10 has more cycles. Next, we apply the model (2) to all the TCRCs and we get the fPCs. We will use this expansion for the discussion below. Figure A.1 in the appendix displays the first three estimated functional principal components for MOA group 1 (top left), MOA group 10 (top right), and for all the chemicals (bottom) for concentration levels high, medium, low, and for the smooth errors.

**Figure 3:**
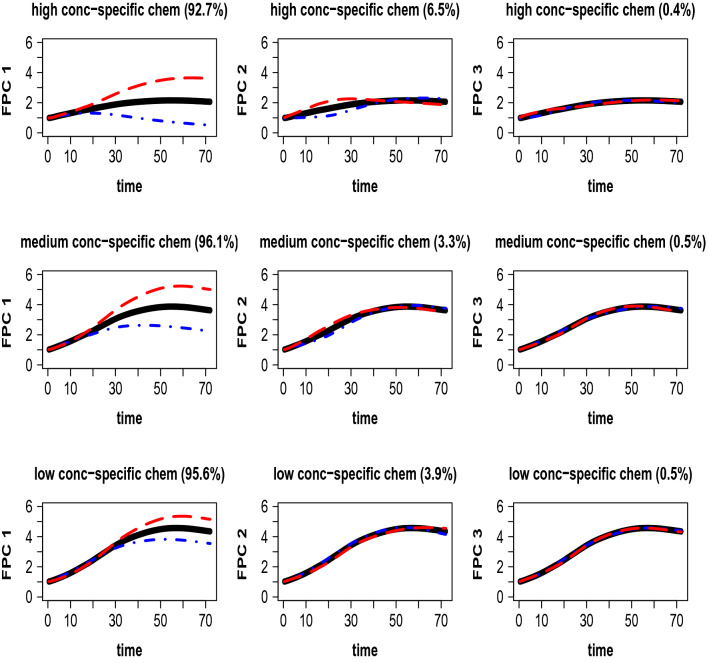
Concentration level-specific means (black, -) plus (red, –) and minus (blue, -.-) a suitable multiple of the first three principal components of $B_{\tau c}$. The respective proportion of variability induced by chemical effects within concentration is given in brackets.

The decomposition of chemical effects $B_{\tau c}$ on fPCs shows where in the TCRCs variability occurs between chemicals. For each concentration $\tau$, the estimated first three principal components of $B_{\tau c}$ are depicted in Fig. 3. To emphasize the information given by the corresponding direction (fPC), we show the effect of adding and of subtracting the estimated principal components multiplied by the square root of the respective eigenvalue to the concentration-specific mean. For high and medium concentration levels, the difference shows up earlier because the red curve and the blue curve of the first and second principal component split away earlier than for weak concentration level. The third principal component in each concentration level does not carry much information.

The proportion in bracket represents the proportion of variability induced by corresponding concentration-specific chemical effects. The variability explained by the fPCs differs between concentrations, but for each concentration level the first principal component explains most variability. We chose the first three fPCs for each concentration-specific fixed chemical effects because they carry almost all the variability induced by chemicals and if we truncate the expansion to include only the first 3 fPCs we obtain an accurate approximation of the experimental TCRCs.

**Figure 4:**
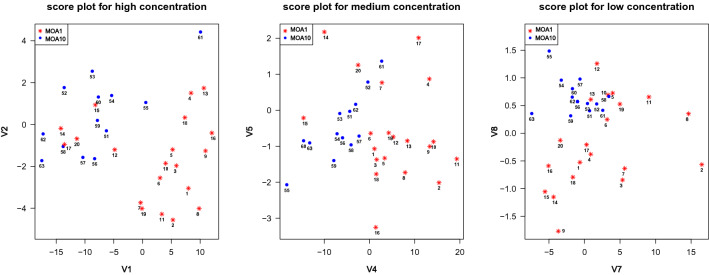
Score plots for the concentration levels. Red stars represent chemicals in MOA group 1 and blue dots those in MOA group 10.

The scores for *high* concentration are termed as V1, V2, V3, the scores for *medium* concentration as V4, V5, V6 and the scores for *low* concentration as V7, V8, V9. We illustrate the scores corresponding to the first two principal components in the two dimensional representation in Fig. 4. The numbers are associated to chemicals as in Table A.1 in the appendix.

**Table 1:** Four MANOVA approximate F statistics (approx F), the numerator degrees of freedom (num DF), the denominator degrees of freedom (den DF), and the P-values (Pr(>F)) used to test for differences among fitted coefficients in the fPCA-based mixed effects model.

Test statistic	Approx F	Num DF	Den DF	Pr(>F)
Wilks	0.25	9	24	2.23e−05
Pillai	3.00	9	24	2.23e−05
Hotelling–Lawley	0.75	9	24	2.23e−05
Roy	3.00	9	24	2.23e−05

For high concentration level, there is an apparent segmentation, but the V1 and V2 scores for five chemicals from MOA group 1 are mixed with the score of chemicals in MOA group 10, and the scores for FAKInhibitor14 (chemical-61 from MOA group 10) are almost on the boundary between the two groups. For medium concentration level, the first fPC can be used to separate the two groups. Excepting 3 chemicals, the values of V4 for chemicals in MOA group 1 are positive. On the other hand, for MOA group 10 the values of V4 are negative, excepting again the chemical FAKInhibitor14. Looking also at the first fPC displayed at the bottom of Fig. A.1 in the appendix, we can deduce that at a medium level of concentration, chemicals in MOA group 1 have a more toxic effect on the cells’ population than chemicals in MOA group 10. For low concentration level, it is not obvious how the scores V7 for the first fPC and V8 for the second fPC are different for MOA group 1 and 10.

Testing the difference between MOA group 1 and MOA group 10 is equivalent to testing if V1 through V9 in MOA group 1 are equal to V1 through V9 in MOA group 10, and we do this applying one-way MANOVA. First we note that the computed scores roughly follow a multivariate Gaussian distribution, which can be checked with the MVN function in the R package MVN. Then, we consider the four standard MANOVA statistics—Wilks, Pillai, Hotelling–Lawley and Roy—detailed in^24^ among other sources. All of these yield strongly significant p-values as displayed in Table 1 indicating significant differences among scores for the MOA groups. Thus, these scores can be used for clustering.

### The B-spline model

**Figure 5:**
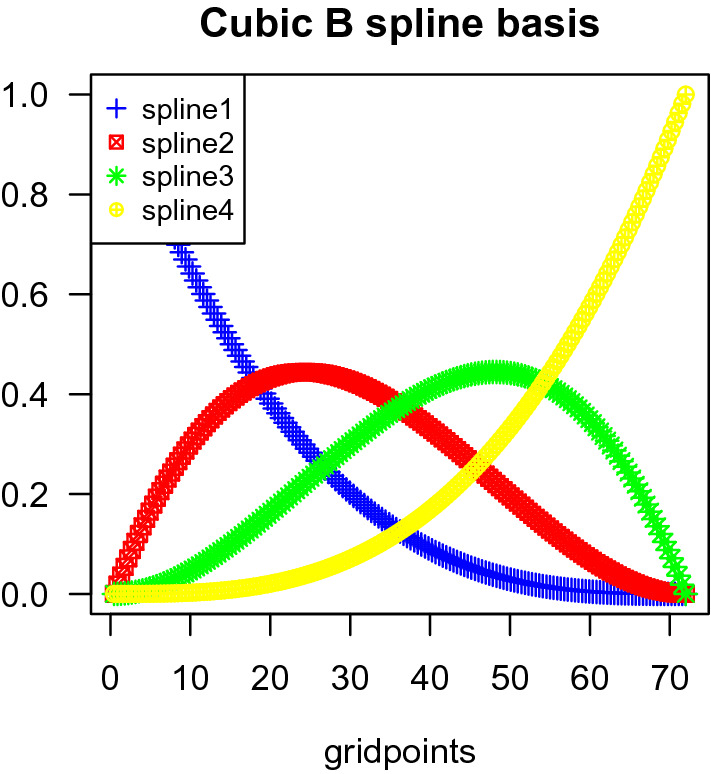
The 4 cubic B-splines.

We can also consider Model 2 projected onto a B-spline basis in order to capture local differences between the MOA types. The TCRCs are smooth curves and they do not have many local minimum and maximum or inflection points, so we split the time interval from 0 to 72 h evenly into four intervals, and we use 4 cubic B-splines denoted $\phi _i$ for $i=1,2,3,4$ (see Fig. 5). The resulting problem can be treated as a multivariate linear mixed effects model.

First, the fixed effects and random effects are represented as a linear combination of spline basis functions:

3 $$\begin{aligned} Y_{\tau cok}(t) = \mu (t) + \sum _{i=1}^4\beta _{\tau ki}\phi _i(t) + \sum _{i=1}^4u_{\tau ci}\phi _i(t) + \sum _{i=1}^4w_{\tau coi}\phi _i(t) + \epsilon (t) \end{aligned}$$

where $Y_{\tau cok}(t)$ denotes the NCI at time $t=1,\ldots , 72$ h and the indices are $\tau$ for concentration, *c* for chemical, *o* for replicate, and *k* for MOA group. For each chemical *c* and MOA group *k*, we vectorize $Y_{\tau cok}(t)$, $\phi _i(t)$, and $\epsilon (t)$ in terms of *t*, *o*, and $\tau$ to get the following format

4 $$\begin{aligned} {\textbf{y}}_{ck} = \varvec{\mu } + X\varvec{\beta }_{k} + Z_1{\varvec{u}}_{c} + Z_2{\varvec{w}}_{c} + \varvec{\epsilon }. \end{aligned}$$

Here ${\textbf{y}}_{ck}$ is a vector with components $Y_{\tau ock}(t), t=1,\ldots , 72, \tau =1,\ldots ,3,$ with $o= 1,\ldots ,4 \text { for }\tau = 1,2 \text { and } o = 1,\ldots ,3 \text { for }\tau = 3$, *X*, $Z_1$, $Z_2$ are design matrices expressed in terms of the cubic B-splines $\phi _i(t)$, $i=1,\ldots , 4$, $t=1,\ldots , 72$, $\varvec{\mu }=(\mu (1),\ldots , \mu (72))^T$ is the global mean, the concentration fixed effects $\varvec{\beta }_k$ are vectors with components $\beta _{\tau ki}$, $\tau =1,\ldots ,3,i=1,\ldots ,4,$ the chemical random effects ${\varvec{u}}_c$ are vectors with components $u_{\tau ci}$, $\tau =1,\ldots ,3,i=1,\ldots ,4,$ and the replication random effects ${\varvec{w}}_{c}$ are vectors with components $w_{\tau coi}$, $i=1,..,4$, $\tau =1,\ldots ,3$, with $o= 1,\ldots ,4 \text { for }\tau = 1,2 \text { and } o = 1,\ldots ,3 \text { for }\tau = 3$. We assume that ${\varvec{u}}$ and ${\varvec{w}}$ are mutually independent.

**Figure 6:**
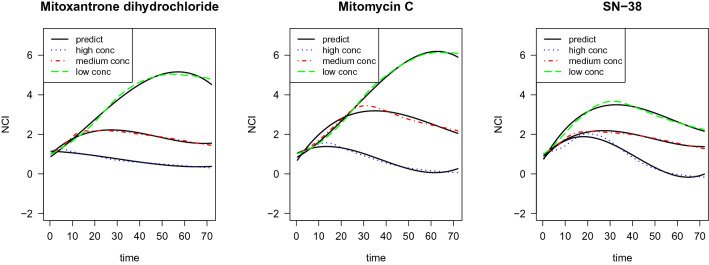
Experimental TCRCs (black plain line) versus predict TCRCs (colored dashed lines).

We estimate the coefficients using the package lme4 in R, and we use the estimated coefficients to reconstruct the TCRCs. Figure 6 shows three chemicals as examples. Black plain line curves correspond to the average of replications of a specific concentration, while the colored dashed line curves are predicted TCRCs without using replication effects. Comparing the fitted and the experimental curves shows that linear mixed effect model with B-spline basis perform well in capturing the overall features of the TCRCs.

**Table 2:** The seven significant differences between fitted B-spline coefficients for MOA group 1 and 10.

Fixed effects	Estimate	Std. error	t value	Pr($>|t|$)
${\hat{\beta }}_{1,10,2}-{\hat{\beta }}_{1,1,2}$	− 1.20	0.32	− 3.75	0.0003
${\hat{\beta }}_{2,10,2}-{\hat{\beta }}_{2,1,2}$	− 0.84	0.32	− 2.65	0.009
${\hat{\beta }}_{1,10,3}-{\hat{\beta }}_{1,1,3}$	2.85	0.66	4.31	0.00004
${\hat{\beta }}_{2,10,3}-{\hat{\beta }}_{2,1,3}$	2.64	0.66	3.98	0.00014
${\hat{\beta }}_{1,10,4}-{\hat{\beta }}_{1,1,4}$	1.74	0.46	3.79	0.00026
${\hat{\beta }}_{2,10,4}-{\hat{\beta }}_{2,1,4}$	1.61	0.46	3.50	0.00071

After estimating all of the coefficients in Model 3 we identify six significant differences between the two MOA groups in Table 2 with a false discovery rate of 0.1 through use of the Benjamini–Yekutieli procedure, which was chosen over the standard Benjamini–Hochberg procedure because these hypothesis tests are not all independent of each other^25^.These are ${\hat{\beta }}_{1,10,2}-{\hat{\beta }}_{1,1,2}$, ${\hat{\beta }}_{2,10,2}-{\hat{\beta }}_{2,1,2}$, ${\hat{\beta }}_{1,10,3}-{\hat{\beta }}_{1,1,3}$, ${\hat{\beta }}_{2,10,3}-{\hat{\beta }}_{2,1,3}$, ${\hat{\beta }}_{1,10,4}-{\hat{\beta }}_{1,1,4}$, and ${\hat{\beta }}_{2,10,4}-{\hat{\beta }}_{2,1,4}$.

A complete list of the estimated coefficients is displayed in Table A.2 in the appendix. We can conclude that chemicals in MOA 1 and MOA 10 show larger difference when the solution concentration level is $\tau =1,2$ being *high* and *medium*, respectively. For both concentration levels, the second, third and fourth basis functions are directions that correspond to significant cluster differences. According to the estimates of significant terms, we can see the coefficients on the second spline of MOA 10 are less than those of MOA 1 indicating that the cells exposed to the chemicals in MOA 1 at the beginning grow faster than the cells exposed to the chemicals in MOA 10. But on the last two splines, the coefficients of MOA 10 are much larger. We conclude that chemicals in MOA 1 kill the cells faster in the second half of the time span.

## Cluster analysis

We first concatenate each set of 11 curves per chemical into one long vector and then use a wavelet decomposition to cluster based on the estimated wavelets coefficients. This approach follows from the use of wavelets for MOA classification in^4^. Wavelet levels 1,...,6 were considered. SOMs-based clustering was applied to the wavelet coefficients and exhaustively tested over all parameter settings for the SOMs algorithm. The best result for clustering the experimental data in MOA group 1 and MOA group 10 had an accuracy of 85.3% and for three cluster separation was 73.3%. In^4^ support vector machines and neural networks were applied to the wavelets coefficients of the same data and the classification rate was more than 90% for binary classification and over 80% for three classes. However, in contrast to the unsupervised approach used for clustering with SOMs, for the classification methods used in^4^ additional information about the data in the training sets is available.

We note that some parameter choices for SOMs resulted in very poor performance and the best performance regardless of wavelet level was to choose the bubble neighborhood function, the rectangular topology, and a toroidal structure. Without prior knowledge of the correct MOA groups, it could be hard to choose the SOMs parameters to ensure good clustering performance.

In the following subsections, we will use the functional mixed effects models from above to project the TCRCs onto a finite basis in order to proceed with multivariate clustering.

### fPCA model clustering

**Figure 7:**
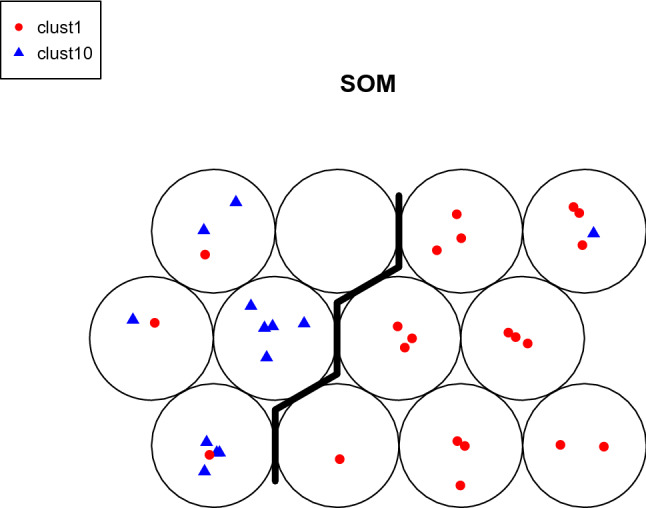
fPCA-based clustering results by SOM.

Applying the standard k-means algorithm to the score vectors (V1,...,V9) we obtain a clustering accuracy of 85.3%, which comes from 4 chemicals from MOA 1 misidentified and 1 chemical from MOA 10 misidentified. However, by looking at Fig. A.1 in the appendix, we notice that many of the fPCs at different concentration levels are very similar and differences between the groups seems to be more evident at medium and high concentration levels. Hence, we remove redundancy in the features used for clustering and we consider subsets of V1–V9. Using just V2 and V4 and repeating application of the k-means algorithm, the clustering accuracy has increased marginally to 88.2%. This agrees with the conclusions of the discussion of the plots in Fig. 4 and indicates that the two most informative directions to consider for clustering are the second fPC for high concentration and first fPC for medium concentration.

As an alternative to k-means, SOMs can be applied to the scores in V1,...,V9. For certain choices of parameters in the SOMs algorithm, clustering accuracy is 88.24%, the same as with k-means; specifically, the best accuracy was achieved via the bubble neighborhood function and the toroidal structure (see Fig. 7). However, other choices of SOMs parameters led to poorer performance. Details are displayed in Table A.3 in the appendix.

### B-spline model clustering

Instead of projecting onto fPCs for the sake of clustering, we can instead project onto the cubic B-spline bases as in section The B-spline model”. For clustering we have to eliminate the group indices *k* from model (4) and use the model

$$\begin{aligned} {\textbf{y}}_{c} = X\varvec{\beta }+Z_1{\varvec{u}}_{c} +Z_2{\varvec{w}}_{c}+\varvec{\epsilon }. \end{aligned}$$

We fit this model to the experimental data in MOA groups 1 and 10 and we use the coefficients of chemical effects ${\varvec{u}}_{c}$ for clustering. Based on the seven significant differences detailed above in Table 2, we tried k-means for subsets of the components of ${\varvec{u}}_{c}$ and we achieved an accuracy rate of 85.3%, similar to the above fPCA-based approach. However, while more than one choice of variables leads to the same classification accuracy, the ratio of between-sum-of-squares to total-sum-of-squares (BSS/TSS) varies quite drastically. The tightest clusters are achieved by clustering with the 4th B-spline for concentration levels high and *medium* indicating that the latter time period for the TCRCs is most critical for determining the difference between MOA groups 1 and 10. These results are displayed in Table 3

**Table 3:** The accuracy rate and between to total sum of squares ratio for k-means clustering after variable selection for three different choices of selected variables.

Splines for clustering	Dimension	Accuracy (%)	BSS/TSS (%)
$\{\phi _4,\textrm{conc}=\text {``high''}\}$, $\{\phi _4,\textrm{conc}=\text {``medium''}\}$	2	85.29	74.9
$\{\phi _2,\textrm{conc}=\text {``high''}\}$, $\{\phi _4,\textrm{conc}=\text {``medium''}\}$	2	85.29	45.0
$\{\phi _3,\textrm{conc}=\text {``low''}\}$, $\{\phi _4,\textrm{conc}=\text {``medium''}\}$	2	85.29	48.5

**Table 4:** Results of binary clustering for B-spline coefficients.

	Assigned labels			Assigned labels		
	A	B		A	B	
MOA 1	17	4	MOA 1	16	5	
MOA 10	1	12	MOA other	2	29	
			Accuracy rate: 85.29%			Accuracy rate: 86.54%
			Assigned labels			Assigned labels

	Assigned labels			Assigned labels		
	A	B		A	B	C
MOA 10	11	2	MOA 1	16	5	0
MOA other	6	25	MOA 10	0	12	1
		Accuracy rate: 81.82%	MOA other	2	6	23
			Accuracy rate: 78.46%			

**Figure 8:**
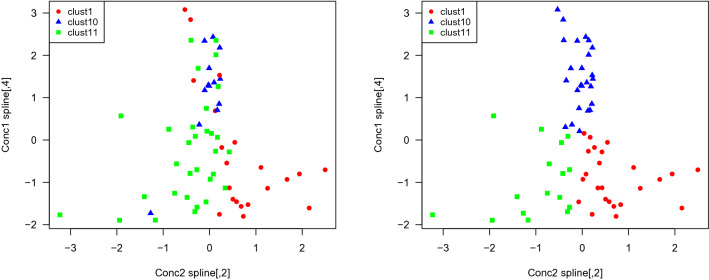
On the left, the true clusters for MOA groups 1, 10, and the remainder. On the right, the results of k-means clustering.

Binary clustering was also carried out for each of MOA groups 1 and 10 to the remaining combined set of 30 TCRCs comprising the other 8 MOA groups. For differentiating MOA 1 from the remainder, it was spline $\phi _2$ at medium concentration and spline $\phi _4$ at low concentration that indicated the significant differences. In contrast, for differentiating MOA 10 from the remainder, it was spline $\phi _2$ at medium concentration paired with spline $\phi _4$ at high concentration. Attempting to cluster MOA 1, 10, and the remainder group simultaneously yielded spline $\phi _4$ at concentrations high and low as well as spline $\phi _2$ at concentration medium as the most significant basis functions to project onto. The three clusters are displayed in Fig. 8. All of the clustering results are detailed in Table 4.

**Figure 9:**
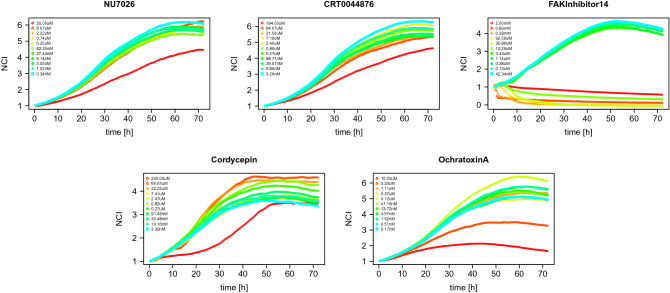
The five chemicals that defy all clustering methods.

### Misidentified chemicals

Regardless of method, the best two-cluster accuracy is consistently 85.3% resulting from one misidentified chemical from MOA 10 and 4 misidentified chemicals from MOA 1, which are displayed in Fig. 9. This is a consequence of the experiment itself. For each chemical, the concentration effect is not known a priori. Hence, researchers had to make educated guesses at the correct chemical concentrations to apply. In some cases, the experiment failed such as for the FAKInhibitor14 from MOA group 10 in the top right of Fig. 9 where all of the cells quickly died for the top 6 concentration levels. In the case of the 4 chemicals from MOA 1, the concentration was not large enough to kill the cells.

## Conclusion

Assessing a chemical’s toxicity is of critical importance for guiding environmental regulations. In this work, we have seen that functional mixed effects models provide a powerful tool for modeling TCRC data and interpreting where the differences between MOA groups lie. Furthermore, these differences can motivate unsupervised clustering of the TCRCs into MOA groups with high accuracy. This clustering task is of particular interest to applied researches as understanding how TCRCs differ among MOA groups will allow chemical cytotoxicity testing to be streamlined in order to target those differences.

## Supplementary Information

Supplementary Information.

## Data Availability

The data that support the findings of this study are available from the Alberta Centre for Toxicology but restrictions apply to the availability of these data, which were used under license for the current study, and so are not publicly available. Data are however available from the authors upon reasonable request and with permission of the Alberta Centre for Toxicology. Please contact the corresponding author, Dr. Adam Kashlak, at kashlak@ualberta.ca.
