# Supplementary material for: Functional non-parametric mixed effects models for cytotoxicity assessment and clustering
Source: Sci Rep. 2023 Mar 11;13:4075. doi: 10.1038/s41598-023-31011-1 (PMC10008646; doi:10.1038/s41598-023-31011-1)
Supplement: Supplementary file 1 — Supplementary Information. [file 41598_2023_31011_MOESM1_ESM.pdf]

Appendix for Functional non-parametric mixed effects models for cytotoxicity assessment and clustering

Tiantian Ma[1], Dan Richard[1,2], Yongqing Yang[1], Adam Kashlak[1,*], Cristina Anton[2]

[1] Mathematical & Statistical Sciences, University of Alberta, Edmonton, Canada
[2] Mathematics & Statistics, Grant MacEwan University, Edmonton, Canada
[*] Corresponding Author

Appendix A. Additional Figures and Tables

Table A.1 lists the names and applied concentrations of all 63 chemicals in the data set. The experiment began by applying the highest concentration chosen by the researchers to a cell culture. This was then diluted by 1/3 for the subsequent culture continuing for 10 dilutions to achieve the 11 original concentration levels. The maximal concentration was intended to be chosen to be just large enough to kill off all of the cells in the culture. The chemicals in this table are separated into MOA groups indicating the cell structure or function targeted by that class of chemicals.

Table A.1: A list of chemicals in MOA groups I - X and maximal/minimal concentrations.

	Group I: DNA/RNA-Nucleic Acid Targets	Concentration(1:3)
1	5-Fluorouracil (5-FU)	200 μM to 3.39 nM
2	Gemcitabine HCl	1650 μM to 27.94 nM
3	Etoposide phosphate	200 μM to 3.39 nM
4	Doxorubicin	100 μM to 1.69 nM
5	Merbarone	200 μM to 3.39 nM
6	Clofarabine	25 μM to 0.42 nM
7	Hydroxyurea	10 μM to 169 nM
8	SN-38	200 μM to 3.39 nM
9	Topotecan	95 μM to 1.61 nM

10	Irinotecan	160 μM to 2.71 nM
11	Cytosine	8950 μM to 151.57 nM
12	ABT-888	308 μM to 5.22 nM
13	Mitoxantrone dihydrochloride	150 μM to 2.54 nM
14	CRT0044876	194 μM to 3.29 nM
15	NU7026	20 μM to 0.34 nM
16	Mitomycin C	200 μM to 3.39 nM
17	Cordycepin	200 μM to 3.39 nM
18	Actinomycin D	2 μM to 0.0339 nM
19	Cisplatin H2O	μM to 2.54 nM
20	Ochratoxin A	10 μM to 0.17 nM
	Group II: Transport Protein-Primary Active transporter Targets	Concentration(1:3)
21	Brefeldin A	40 μM to 0.68 nM
22	Exo 1	300 μM to 5.08 nM
23	Leptomycin B	20 nM to 0.000339 nM
24	Concanamycin A	0.2 μM to 0.003 nM
25	Thapsigargin	2 μM to 0.0339 nM
26	BHQ	400 μM to 7 nM
	Group III: Protein-Actin Targets	Concentration(1:3)
27	Bafilomycin A1	0.3212 μM to 0.01 nM
28	Cytochalasin D	20 μM to 0.339 nM
29	Cytochalasin B	20 μM to 0.339 nM
30	Latrunculin A	2 μM to 0.0339 nM
31	Latrunculin B	2 μM to 0.0339 nM
	Group IV: Protein-Tubulin Targets	Concentration(1:3)
32	Docetaxel	1 μM to 0.02 nM
33	Paclitaxel	20 μM to 0.339 nM
34	Vincristine Sulfate	250 μM to 4.23 nM
35	Vinblastine sulfate	40 μM to 0.68 nM
	Group V: Ribosome-50S Subunit Targets	Concentration(1:3)
36	Emetine	50 μM to 0.847 nM
37	Puromycin	1000 μM to 17 nM
38	Anisomycin	10 μM to 0.17 nM
	Group VI: Transport Proteins-Electrochemical Potential-driven Transporters	Concentration(1:3)
39	Oligomycin	20 μM to 0.339 nM

40	Antimycin A	200 μM to 3.387 nM
41	Rotenone	200 μM to 3.387 nM
42	CCCP	100 μM to 1.69 nM
	Group VII: Ion Channel Targets	Concentration(1:3)
43	Valproic acid	50 mM to 847 nM
44	BAPT-am	60 μM to 1 nM
	Group VIII: Enzyme Targets	Concentration(1:3)
45	Cyclosporin A	100 μM to 1.69 nM
46	FK-506	50 μM to 1 nM
47	(S)-HDAC-42	128 μM to 2.17 nM
48	SAHA	151 μM to 2.56 nM
49	W7 HCl	200 μM to 3.39 nM
	Group IX: Receptors	Concentration(1:3)
50	benzo[a]pyrene	100 μM to 1.69 nM
	Group X: Protein- Motor Targets	Concentration(1:3)
51	Monastrol	100 μM to 1.69 nM
52	S-trityl-cysteine	100 μM to 1.69 nM
53	Dimethylenastron	40 μM to 0.68 nM
54	Y-27632	188 μM to 3.18 nM
55	HA1100 hydrochloride	1000 μM to 16.94 nM
56	Ro32-3555	200 μM to 3.39 nM
57	Batimastat	200 μM to 3.39 nM
58	MLCKInhibPep18	94.5 μM to 1.6 nM
59	Blebbistatin	100 μM to 1.69 nM
60	ML7 hydrochloride	100 μM to 1.69 nM
61	FAKInhibitor14	2500 μM to 42.34 nM
62	PF573228	40 μM to 0.68 nM
63	PF431396	5 μM to 0.08 nM

Table A.2 displays all of the fitted coefficients from the B-spline model of Section 4.2 of the main article. Of note for the cytotoxicity data is the second half of the table, which details the differences in B-spline coefficients between MOA group 10 and group 1. For each of the $\hat{\beta}$'s, the first index corresponds to the concentration level with 1 = *high*, 2 = *medium*, and 3 = *low*. The second index is for MOA group 1 or 10. The third indicates the specific cubic B-spline, which are temporally ordered meaning that 1 is for the beginning of the experiment whereas 4 is for the end. Glancing at the p-values, we see that B-spline 1 does not yield significant differences

between the MOA groups mainly because all cell cultures began from the same starting point. Meanwhile, B-splines 3 and 4 at high and medium concentration yield significant differences. At low concentration, MOA group differences are not noticeable.

Table A.2: All of the estimated coefficients from the functional mixed effects model using B-splines.

fixed effects:	Estimate	Std. Error	t value	Pr(> $\|t\|$)
$\hat{\mu}$	3.75	0.28	13.19	<2e-16
$\hat{\beta}_{1,1,1}$	-2.79	0.29	-9.80	3.35e-16
$\hat{\beta}_{2,1,1}$	-2.88	0.29	-10.09	< 2e-16
$\hat{\beta}_{3,1,1}$	-2.89	0.29	-10.11	< 2e-16
$\hat{\beta}_{1,1,2}$	-0.88	0.35	-2.56	0.011
$\hat{\beta}_{2,1,2}$	-0.40	0.35	-1.16	0.250
$\hat{\beta}_{3,1,2}$	-0.80	0.35	-2.32	0.022
$\hat{\beta}_{1,1,3}$	-2.29	0.50	-4.60	8.09e-06
$\hat{\beta}_{2,1,3}$	-0.24	0.50	-0.49	0.626
$\hat{\beta}_{3,1,3}$	1.36	0.50	2.73	0.007
$\hat{\beta}_{1,1,4}$	-2.12	0.40	-5.28	8.18e-07
$\hat{\beta}_{2,1,4}$	-1.04	0.40	-2.60	0.011
$\hat{\beta}_{1,10,1} - \hat{\beta}_{1,1,1}$	-0.04	0.04	-0.98	0.330
$\hat{\beta}_{2,10,1} - \hat{\beta}_{2,1,1}$	0.05	0.04	1.08	0.284
$\hat{\beta}_{3,10,1} - \hat{\beta}_{3,1,1}$	0.04	0.05	0.85	0.399
$\hat{\beta}_{1,10,2} - \hat{\beta}_{1,1,2}$	-1.20	0.32	-3.75	0.0003
$\hat{\beta}_{2,10,2} - \hat{\beta}_{2,1,2}$	-0.84	0.32	-2.65	0.009
$\hat{\beta}_{3,10,2} - \hat{\beta}_{3,1,2}$	-0.27	0.32	-0.85	0.399
$\hat{\beta}_{1,10,3} - \hat{\beta}_{1,1,3}$	2.85	0.66	4.31	4.03e-05
$\hat{\beta}_{2,10,3} - \hat{\beta}_{2,1,3}$	2.64	0.66	3.98	0.00014
$\hat{\beta}_{3,10,3} - \hat{\beta}_{3,1,3}$	1.39	0.66	2.10	0.0386
$\hat{\beta}_{1,10,4} - \hat{\beta}_{1,1,4}$	1.74	0.46	3.79	0.00026
$\hat{\beta}_{2,10,4} - \hat{\beta}_{2,1,4}$	1.61	0.46	3.50	0.00071
$\hat{\beta}_{3,10,4} - \hat{\beta}_{3,1,4}$	0.67	0.46	1.47	0.145

Table A.3: The performance of SOMs on the fPCA coefficients under different parameter settings for the SOMs algorithm.

neigh_func	topology	structure	grid	accuracy rate
bubble	hexagonal	Planar	6×5	79.41%
			4×3	**88.24%**
		Toroidal	6×5	**88.24%**
			4×3	**88.24%**
	rectangular	Planar	6×5	64.71%
			4×3	**88.24%**
		Toroidal	6×5	**88.24%**
			4×3	**88.24%**
Gaussian	hexagonal	Planar	6×5	**88.24%**
			4×3	82.35%
		Toroidal	6×5	85.29%
			4×3	82.35%
	rectangular	Planar	6×5	67.65%
			4×3	82.35%
		Toroidal	6×5	82.35%
			4×3	82.35%

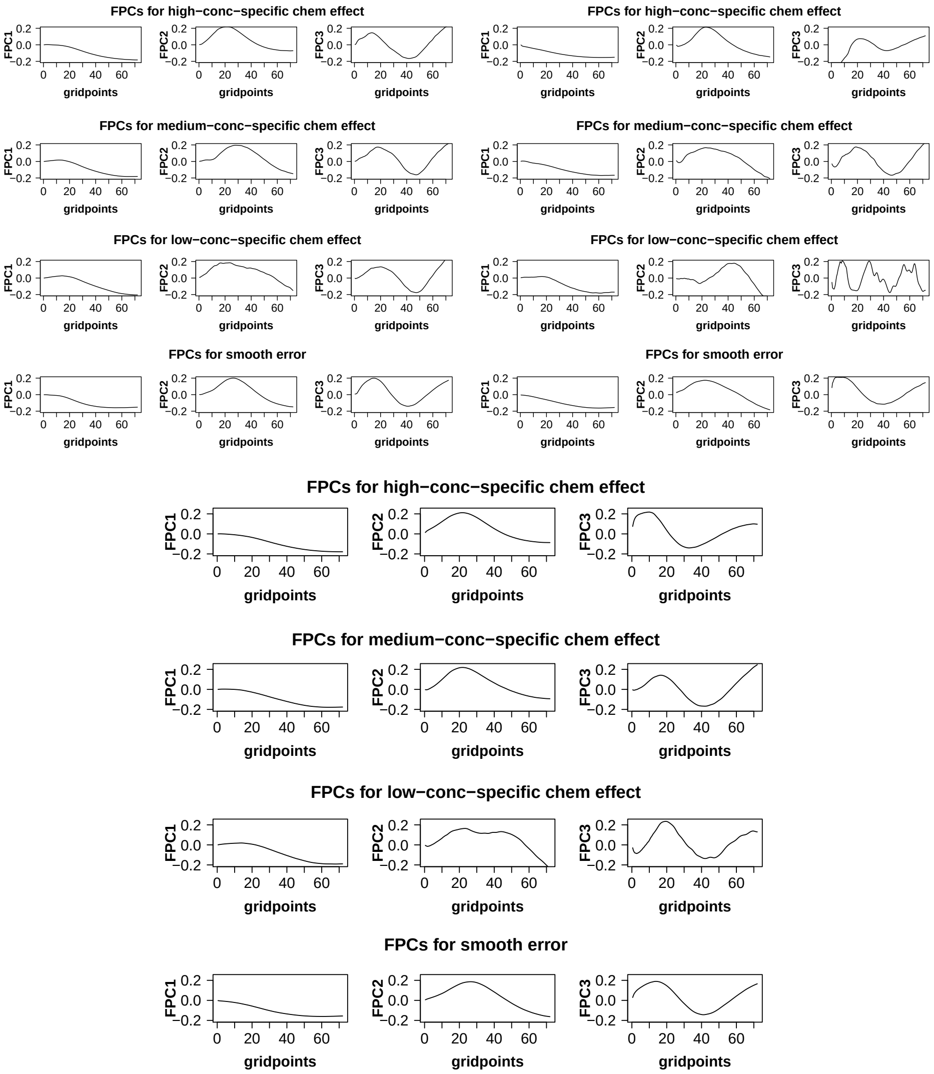

Figure A.1: The first three estimated functional principal components for MOA group 1 (top left), MOA group 10 (top right), and for all the chemicals (bottom) for concentration levels high, medium, low, and for the smooth errors.
